# Tuber shape and eye depth variation in a diploid family of Andean potatoes

**DOI:** 10.1186/s12863-015-0213-0

**Published:** 2015-05-30

**Authors:** Hannele Lindqvist-Kreuze, Awais Khan, Elisa Salas, Sathiyamoorthy Meiyalaghan, Susan Thomson, Rene Gomez, Merideth Bonierbale

**Affiliations:** International Potato Center (CIP), Av. La Molina 1895, Apartado 1558, Lima 12, Peru; The New Zealand Institute for Plant & Food Research Limited, Private Bag 4704, Christchurch, 8140 New Zealand

**Keywords:** Tuber shape, Eye depth, Morphological descriptors, Quantitative trait loci (QTL)

## Abstract

**Background:**

Tuber appearance is highly variable in the Andean cultivated potato germplasm. The diploid backcross mapping population ‘DMDD’ derived from the recently sequenced genome ‘DM’ represents a sample of the allelic variation for tuber shape and eye depth present in the Andean landraces. Here we evaluate the utility of morphological descriptors for tuber shape for identification of genetic loci responsible for the shape and eye depth variation.

**Results:**

Subjective morphological descriptors and objective tuber length and width measurements were used for assessment of variation in tuber shape and eye depth. Phenotypic data obtained from three trials and male–female based genetic maps were used for quantitative trait locus (QTL) identification. Seven morphological tuber shapes were identified within the population. A continuous distribution of phenotypes was found using the ratio of tuber length to tuber width and a QTL was identified in the paternal map on chromosome 10. Using toPt-437059, the marker at the peak of this QTL, the seven tuber shapes were classified into two groups: cylindrical and non-cylindrical. In the first group, shapes classified as ‘compressed’, ‘round’, ‘oblong’, and ‘long-oblong’ mainly carried a marker allele originating from the male parent. The tubers in this group had deeper eyes, for which a strong QTL was found at the same location on chromosome 10 of the paternal map. The non-cylindrical tubers classified as ‘obovoid’, ‘elliptic’, and ‘elongated’ were in the second group, mostly lacking the marker allele originating from the male parent. The main QTL for shape and eye depth were located in the same genomic region as the previously mapped dominant genes for round tuber shape and eye depth. A number of candidate genes underlying the significant QTL markers for tuber shape and eye depth were identified.

**Conclusions:**

Utilization of a molecular marker at the shape and eye depth QTL enabled the reclassification of the variation in general tuber shape to two main groups. Quantitative measurement of the length and width at different parts of the tuber is recommended to accompany the morphological descriptor classification to correctly capture the shape variation.

**Electronic supplementary material:**

The online version of this article (doi:10.1186/s12863-015-0213-0) contains supplementary material, which is available to authorized users.

## Background

Large variability in tuber appearance exists in the Andean cultivated potato germplasm. While wild potatoes are generally small and round with rather superficial eyes [1] the tubers of early-domesticated landrace cultivars are larger and have great variability in shape and eye depth [2]. Andean farmers seek to maintain this rich diversity by cultivating mixtures of landraces in their fields. Cultural rites, folk nomenclature, and the association of certain shapes with distinct culinary preferences and uses have been passed on through generations [2]. Thus, the user preferences that still persist in the original home of potato domestication are very distinct from those associated with potato in the areas that adopted this crop relatively recently. In modern varieties, superficial eyes, uniform color and round or oblong shape are preferential as these facilitate handling and processing. Heavy selection has consequently led to low diversity for these traits in modern cultivars.

For the purpose of revealing the genetic control of tuber shape and eye depth, various classification categories have been used, reflecting the variability present. Some studies featuring *Solanum tuberosum* (at either tetraploid or diploid level), have used two (round, long), three (round, oval and long), or four (round, oval, long oval, very long oval) shape categories [3–11]. In other cases, such as in a hybrid population between *S. verrucosum* and *S. microdontum,* six shape types were recorded (long, long oval, oval, round oval, round, compressed) [12]. In a *S. phureja* derived population, initially eight shape types were scored, but for genetic mapping they were grouped into three main classes (round, oval, long) [13, 14]. Eye depth has previously been recorded using three [10, 14, 15] to nine classes [11–13].

The International Potato Center (CIP) currently holds the largest biodiversity of cultivated potatoes. The morphological descriptors for tuber shape used by the CIP genebank include eight basic categories: compressed, round, ovoid, obovoid, elliptic, oblong, long-oblong and elongated, as well as an additional set of nine unusual shapes (for example paw-like). For eye depth, the scale has five classes: protuberant, superficial, slightly deep, deep and very deep [16].

The diploid backcross mapping population ‘DMDD’ was developed at CIP [17] and was used to anchor the first published potato genome sequence of potato (DM) and to develop a dense genetic map [18]. The female grandparent of the population, DM, belongs to the *S. tuberosum* Group Phureja [19] and the recurrent male parent, DI, to *S. tuberosum* diploid Andigenum Group Goniocalyx [20,21], thus providing some of the allelic variation present in the Andean landraces. DMDD segregates for numerous morphological, reproductive, physiological, biochemical and abiotic stress related traits [22, 23], which, combined with the high-density genetic map and the high-quality reference genome sequence, open up great opportunities for trait-QTL and gene discovery studies.

The present work was conducted to evaluate the utility of morphological descriptors for tuber shape to the identification of genetic loci responsible for the shape and eye depth variation. In addition, the reference genome sequence of potato was used to search for potential candidate genes responsible for the QTL effect found.

## Results

### Phenotypic data

**Fig. 1:**
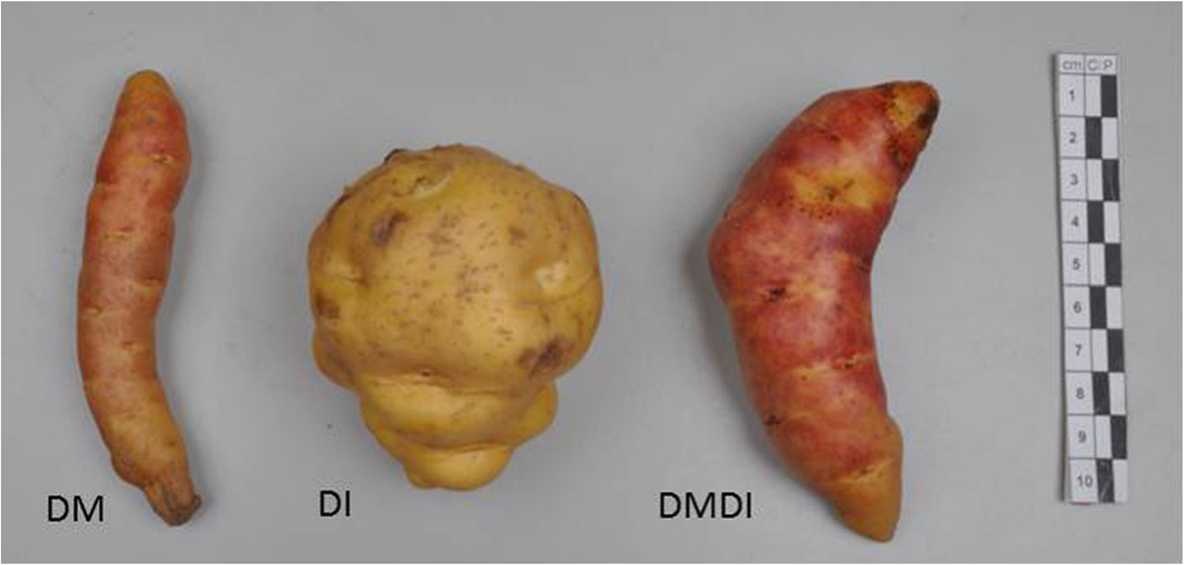
Tuber shape and eye depth phenotypes of the DMDD population progenitors. The female grandparent, DM (CIP 801092), had elongated tubers with superficial eyes; the recurrent male parent, DI (CIP 703825), had round tubers and deep eyes; and DMDI (CIP 305156.17) had elliptic tubers and slightly deep eyes

**Fig. 2:**
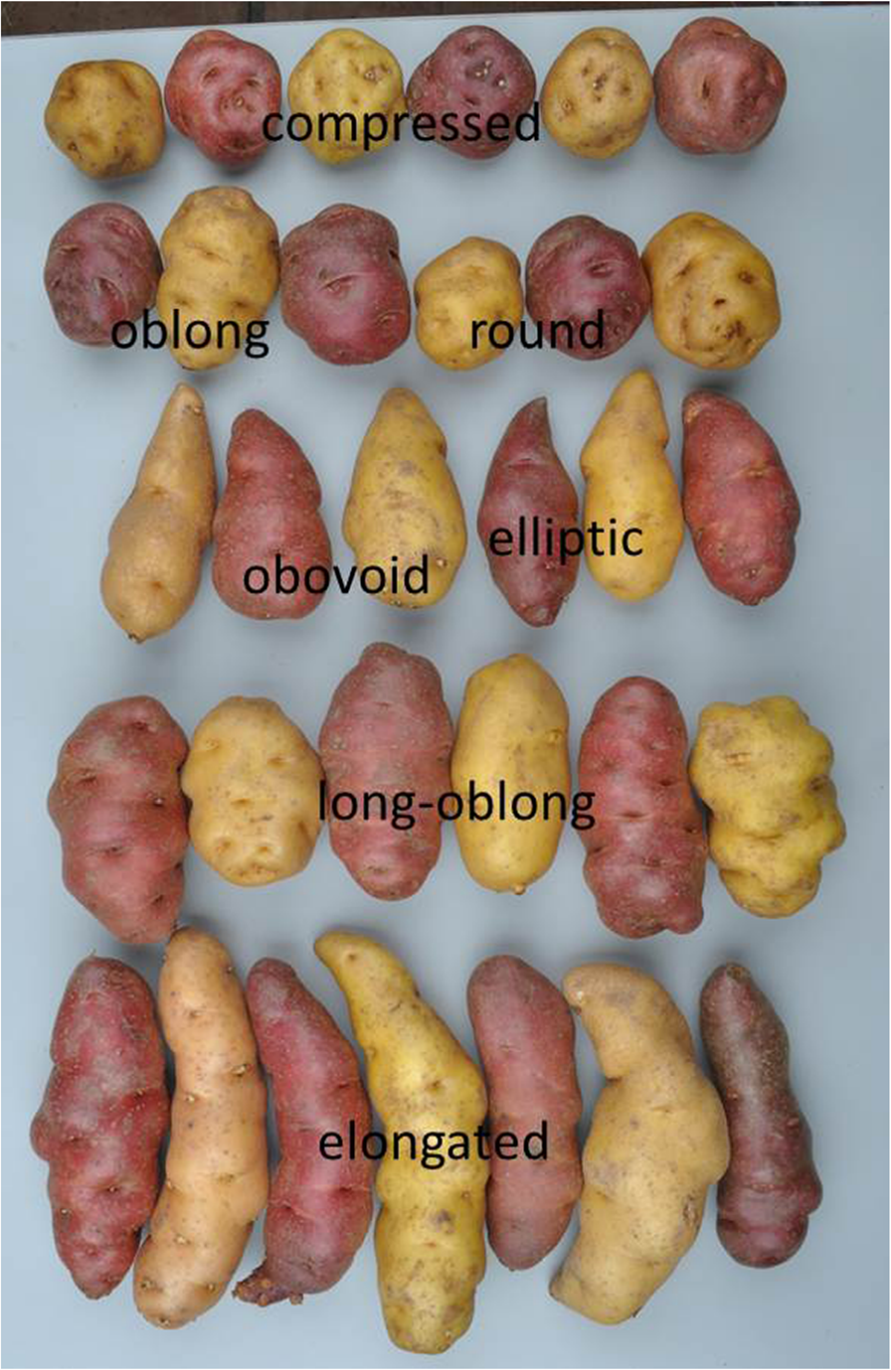
Tuber shape and eye depth variation in the DMDD progeny in the trial field3. Seven different shapes: ‘compressed’, ‘round’, ‘oblong’, ‘obovoid’, ‘elliptic’, ‘long-oblong’ and ‘elongated’, were found in the progeny. The tuber heel is pointing up

**Fig. 3:**
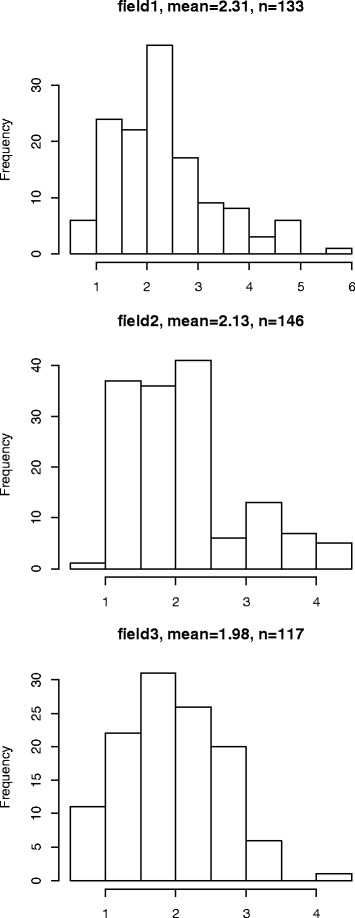
The ratio of tuber length to width distribution in the DMDD progeny in the three field trials (field 1–3)

Among the parental genotypes of DMDD, DM (CIP 801092) had elongated tubers with superficial eyes, DI (CIP 703825) had round tubers and deep eyes, and DMDI (CIP 305156.17) had elliptic tubers and slightly deep eyes (Fig. 1). Seven different shapes were found in the progeny in all trials, the most prevalent categories being obovoid, elliptic, oblong, long-oblong and elongated (Fig. 2, Table 1). The mean values in all experiments were quite similar for the ratio of tuber length to the width, but the distribution was skewed towards the smaller values in field 1 and field 2 trials (Fig. 3). Most progeny individuals had slightly deep or deep eyes, but there were also some individuals with very deep or superficial eyes (Table 1).

**Table 1:** Distribution of DMDD progeny individuals in general tuber shape and eye depth categories in the three field trials

Trial (number of progeny individuals)	General tuber shape							Eye depth			
	Compressed	Round	Obovoid	Elliptic	Oblong	Long-oblong	Elongated	Superficial	Slightly deep	Deep	Very deep
Field1^a^ (n = 134)	0	7	22	27	30	23	25	8	40	54	20
Field2 (n = 148)	1	1	27	26	34	35	24	2	47	74	25
Field3 (n = 117)	10	2	30	3	25	40	7	0	41	75	1

^1^ eye depth was evaluated on only 122 individuals

### Characteristics of parental genetic maps of DMDD

Both parental maps had 12 chromosomes corresponding to the chromosomes of potato (Table 2). For the paternal map, the average chromosome length was 58.7 cM, the average marker interval was 1.9 cM and contained an average of 29 markers giving a total of 339 markers. A total of 423 markers mapped on the maternal map with an average chromosome length of 65.4 cM, an average marker interval of 2.1 cM and an average of 35 markers per chromosome. More details on the genetic map have been described by Khan et al. [23].

**Table 2:** Characteristics of the separate genetic maps of the DMDD population parents

	Chromosome	Length of the linkage group (cM)	Number of markers	Mean marker interval (cM)	Max marker interval (cM)
Paternal map	1	57.71	35	1.70	12.84
	2	64.30	44	1.50	21.36
	3	59.88	26	2.40	16.52
	4	73.65	26	2.95	15.83
	5	39.90	14	3.07	14.20
	6	57.56	23	2.62	9.15
	7	60.45	31	2.02	18.20
	8	54.15	24	2.35	17.25
	9	72.31	45	1.64	12.86
	10	62.29	23	2.83	21.47
	11	43.21	26	1.73	7.37
	12	59.01	22	2.81	10.56
	Total	704.41	339		
Maternal map	1	85.11	66	1.31	15.48
	2	63.07	39	1.31	12.49
	3	87.37	52	1.71	24.73
	4	85.13	30	2.94	19.81
	5	54.82	10	2.51	12.75
	6	63.31	39	1.67	5.47
	7	66.80	29	2.39	14.16
	8	55.30	34	1.68	11.14
	9	34.22	27	1.27	8.42
	10	64.99	53	1.25	9.87
	11	57.34	29	2.05	10.49
	12	67.55	15	4.82	22.99
	Total	784.99	423		

### Tuber shape and eye depth QTL

**Fig. 4:**
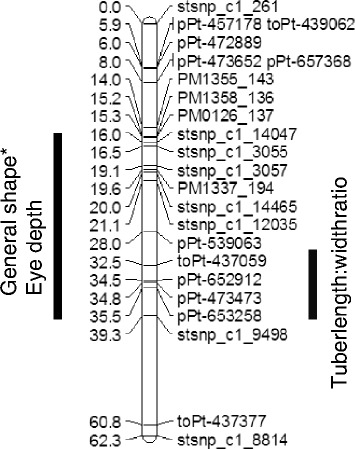
Schematic representation of the QTL detected in chromosome 10 of the paternal genetic map. The marker locations on the linkage map are shown as cumulative distances in cM. The right side of the linkage map shows the QTL for the ratio of tuber length to width obtained by interval mapping and the left side shows the Kruskall-Wallis confidence limits at p < 0.001 for the eye depth and general shape QTL. *QTL for general shape was detected only in trial field1

**Fig. 5:**
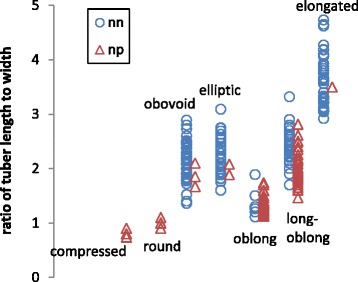
The occurrence of the QTL peak marker toPt-437059 genotypes nn and np in categorical tuber shape classes

QTL for the ratio of tuber length to width, general shape and eye depth were all found within the same region of chromosome 10 in the paternal map (Table 3, Table 4, Fig. 4). The largest-effect QTL was found for the ratio of tuber length to width in all experiments, regardless of planting material, growing conditions and environment, and explained between 37 and 40 % of the variation (Table 3). The marker at the peak of this QTL is toPt-437059 and the np allele from the DI parent is associated with the compressed, round, oblong and long-oblong shapes (Table 5). These shape classes have in common a cylindrical shape that has similar width throughout the whole tuber length. The individuals that lack the DI marker allele (thus are nn) have tubers that belong in the classes obovoid, elliptic and elongated, which have a markedly narrower base and/or apex as compared to the middle width (Table 5). The grouping of individuals is rather obvious in every class except for the field 3 trial, where unexpectedly many individuals were scored in the long-oblong class. Figure 5 depicts the overall relationship between the general shape and the ratio of the tuber length to the width.

**Table 3:** QTL significant at 99% genome-wide significance threshold, identified for the ratio of tuber length to width

Map	Trial	LOD	%	2-LOD interval cM	Chromosome	Peak cM	Peak marker
Paternal map	Field3	13.21	40.5	28.95-39.308	10	32.545	toPt-437059
	Field2	15.07	37.8				
	Field1	13.64	37.6				
Maternal map	Field2	3.47	10.4	16.575-40.634	5	32.808	pPt-650026

**Table 4:** Marker intervals with significant associations identified by Kruskal Wallis test for general tuber shape and eye depth

Map	Chromosome	Trait	Trial	Interval cM^a^	Peak cM	Peak marker	K	P-value
Paternal map	10	Eye depth	Field2	16.473-39.308	32.545	toPt-437059	33.018	0.0001
			Field1	16.473-39.308	32.545	toPt-437059	30.75	0.0001
			Field3	16.473-21.097	21.097	stsnp_c1_12035	11.879	0.001
		General shape	Field1	15.221-39.308	27.955	pPt-539063	24.459	0.0001
Maternal map	5	General shape	Field2	23.943-42.069	32.808	pPt-650026	19.501	0.0001
	12	Eye depth	Field2	29.089-44.555	33.693	pPt-459333	16.071	0.0001

^1^ Markers that have the significance level higher or the same as 0.001

**Table 5:** Classification of the DMDD progeny individuals based on the marker toPt-437059 genotype, tuber shape and eye depth in the three field trials

ToPt-437059 genotype/trial		Tuber shape								Eye depth			
		Mean length:width ratio (stdev)	Compressed	Round	Obovoid	Elliptic	Oblong	Long-oblong	Elongated	Superficial	Slightly deep	Deep	Very deep
nn	Field1	2.73 (0.77)	0	0	18	16	1	8	19	0	25	37	0
	Field2	2.53 (0.78)	0	0	19	17	2	6	18	1	30	27	4
	Field3	2.36 (0.52)	0	0	25	3	4	24	6	0	25	37	0
np	Field1	1.6 (0.48)	0	5	1	2	20	13	0	2	2	23	14
	Field2	1.5 (0.39)	1	0	1	0	23	16	0	0	3	24	14
	Field3	1.43 (0.50)	8	2	1	0	18	11	1	0	10	30	1

Only progeny individuals that were included in all experiments (in total 103) are included

The cylindrical tubers have more often very deep eyes compared to the non-cylindrical tubers, but both shape types have deep eyes (Table 5). In the maternal map, a small effect QTL for the ratio of tuber length to width and general shape was found in the same region on chromosome 5 but it was detected only in the trial field 2 (Table 3, Table 4). A QTL for eye depth was also found in chromosome 12 on the maternal map (Table 4).

### Candidate genes

We identified a number of candidate genes underlying the significant QTL for tuber shape and eye depth (Additional file 1: Table S1). Pseudomolecule sequences corresponding to approximately 2 cM at the QTL peaks by interval mapping in chromosomes 5, 10 and 12 were scanned for candidate genes. There were a total of 607, 375 and 133 genes within the regions surrounding the QTL on chromosomes 5, 10 and 12 respectively. Many of the genes found in the QTL regions are annotated as hypothetical proteins and, for example, disease resistance genes. The most interesting gene families from the tuber shape and eye depth perspective belong to gene families associated with cell structure and function, including homeobox, expansin, extension and genes related to the production and modification of pectins (Additional file 1: Table S1). Homeobox genes *POTH1* and a *BEL-1* like gene were found at a distance of 1.94 Mb and 1.37 Mb from the QTL markers pPt-650026 on chromosome 5 and toPt-437059 on chromosome 10 respectively. Three members of the expansin gene family were also found in the region of significant QTL on chromosomes 5 and 10; *β-expansin* and *expansin* were located at a distance of 1.03 Mb and 1.12 Mb respectively from the QTL marker PM0333_219 on chromosome 5, and *α-expansin* was located at a distance of 1.78 Mb from the QTL marker –toPt-437059 on chromosome 10. Three extension genes were also identified within 1.11 Mb of the QTL marker PM0333_219 on chromosome 5. Many genes related to the production and modification of pectins are found in the proximity of QTL markers pPt-650026 and PM0333_219 in chromosome 5, and toPt-437059 in chromosome 10. There are some notable transcription factor genes underlying the major chromosome 10 QTL marker toPt-437059, such as the GRAS transcription factor *SCARECROW*, and the AP2/ERF domain-containing transcription factor, *ERF5*.

## Discussion

Seven different shapes of tubers were found in the DMDD progeny according to the general tuber shape descriptors currently in use at the genebank of the International Potato Center. A continuous distribution of phenotypes was evident for the ratio of tuber length to width and a strong QTL for this trait was identified on chromosome 10 of the paternal map. With the help of the QTL marker toPt-437059, the seven tuber shapes were able to be classified into two groups: cylindrical and non-cylindrical. The shapes compressed, round, oblong and long-oblong, belonging to the first group, carry the marker allele originating from the recurrent male parent; while the shapes obovoid, elliptic and elongated form the second group and carry the marker allele of the female parent. The individuals with the marker allele from the male parent had significantly lower ratio of tuber length to width than the individuals with the female marker allele. The QTL is located in the same genomic region as the previously mapped dominant genes for round tuber shape [9] eye depth [10] and shape QTL [11]. The shape locus *Ro* (round tuber shape) on chromosome 10 is controlled by multiple alleles in *S. tuberosum* [9]. The alleles at the *Ro* locus in the native Andean diploids, such as DMDD progenitors *S. tuberosum phureja* and *S. tuberosum goniocalyx* may differ from those of *S. tuberosum,* resulting in variation not only in the tuber length, but also in the shape of the apex and the base. The shape variation in the native Andean potatoes is impressive, ranging from round to thin, while complicated paw-like and other shape types are also found. In all, there are 17 different tuber shapes recognized as formal morphological descriptors [16] but it is unknown whether all this variation can be explained by different alleles at the *Ro* locus. Recording general tuber shape descriptors in a wider base population and association mapping are required to test this hypothesis. While the major QTL on chromosome 10 plays the most important role in the classification of cylindrical and non-cylindrical tuber shape in DMDD, another minor QTL on chromosome 5 originating from the female parent also has an effect on tuber shape. This is consistent with other studies that report several other minor QTLs accounting for tuber shape variation [11–13, 24–26].

Round shape was found to be linked with deep eyes in studies involving diploid *S. tuberosum x S. phureja* segregating populations [10,11]. In Andean potatoes deep eyes are also found in tubers that are long oblong. In the DMDD progeny, the longer tubers of non-cylindrical type frequently had slightly deep and deep eyes, but tubers with cylindrical shape more often had deep and very deep eyes. Eye depth is influenced by the environment and, in addition, it may be difficult to visually distinguish slightly deep and deep eyes. However, in the DMDD progeny, very deep eyes were never found in the elliptic or obovoid individuals (non-cylindrical type), thus there may be a link between the apex/base shape and eye depth. Studies have suggested a major gene for eye depth in tuberosum based material [10, 14, 15]; however, what is considered deep eyes would not necessarily seem deep in the Andean material.

A single gene, *eyd*, was reputed to largely control eye depth [10, 14, 15]. The *Eyd/eyd* locus was mapped on chromosome 10 and deep eye was reported to be dominant [10]. Maris [15] first noted the link between the eye depth locus and the tuber shape locus. *Ro* and *Eyd* are tightly linked and separated by approximately 4 cM [10]. In our study the LOD interval for the eye depth QTL is wider than that of the ratio for tuber length to width. The interaction of major genes with one or more modifier genes has been suggested as a reason for the intermediate phenotypes such as oval tuber shape and medium deep eyes [10]. Also minor QTL for eye depth have been identified [12, 13].

The effective management and exchange of crop germplasm relies on standard morphological and molecular descriptors. Morphological descriptors are also an essential element of systems for the testing of distinctness, uniformity, and stability (DUS) of new plant varieties. In a study of the practical utility of recommended potato descriptors for DUS of modern potato varieties, general potato shape was rejected as having high coefficient of variation between independent evaluators [27]. Data from qualitative measurements that include tuber width at the rose and heel ends could be used to improve the accuracy of the classification and to capture most of the variation in a bi-parental cross. However, in characterization of landrace collections expressing a much higher level of variability for tuber shape, the descriptors remain of high importance.

The elucidation of the reference potato genome sequence, including the annotation of around 39,000 protein-coding genes [28], has opened many avenues for the research community including the rapid identification of candidate genes underlying trait loci. In the significant QTL for shape and eye depth several gene families associated with cell structure and function in potato tubers were found. Homeobox genes that encode homeodomain proteins are transcription factors for several important genes in development [29] and *KNOX* and *BEL* genes are primary members of the three amino acid loop extension (TALE) superclass of homeobox genes in plants [30]. The partnering of the *BEL-1* like transcription factor *StBEL5* and *KNOX POTH1*, has been shown to have a key role in tuber development [31–33]. Both *BEL-1* and *POTH1* are involved in biosynthesis of gibberellic acid [34], which is an important regulator of tuberization in potato [35]. Recently 14 *BEL1*-like genes in potato, including *StBEL35* on chromosome 10, have been reported [36]. The α-expansins and β-expansins are involved in cell-wall loosening, cell separation and cell expansion in tubers and stems [37,38]. Multiple gene families make up the extensin superfamily, such as the hydroxyproline-rich glycoproteins (HRGPs) which are abundant in cell walls of dicots [39, 40] and have been shown to have an involvement in wound healing in potato tubers [41]. Several studies have demonstrated the role of pectin-related genes in potato tuber development including the regulation of cell adhesion, expansion and differentiation, as well as cell-wall mechanical properties [41–44]. AP2/ERF proteins have been shown to play an important role in the regulation of a variety of biological processes related to growth and development in plants [45–47].

In summary, combining QTL mapping and comparative genomics we have identified a large set of potential candidate genes responsible for tuber shape and eye depth in potato. Fine mapping or association mapping is required to narrow down the candidates for further functional studies and allele mining to implicate genes in potato tuber shape.

## Conclusions

QTL in chromosome 10 largely controls tuber shape and eye depth in the bi-parental cross DMDD, which samples some of the shape variation present in the Andean native potatoes. Molecular marker at this QTL enabled the reclassification of the large variation spanning seven different shape classes to two main groups, cylindrical and non-cylindrical, which reflect the ratio of tuber length to width. Morphological descriptor classification remains important in the characterization of the variable germplasm, but should be accompanied by the quantitative measurement of the length and width at different parts of the tuber to correctly capture the shape variation.

## Methods

### Plant Material, field and greenhouse evaluation

The diploid backcross population DMDD [17,18] ((DM X DI) X DI) was developed by crossing the homozygous DM1-3 516 R44 as female with the heterozygous diploid *S. tuberosum* Stenotomum Group (formerly *S. stenotomum* ssp. *goniocalyx* accession CIP703825 (DI) as male, and backcrossing a resulting hybrid CIP305156.17 (DMDI) again with the DI as male parent. The progeny consists in total of 227 individuals, and of those 180 were used to construct the genetic map. The population was grown in three field trials: La Molina, Lima (field1, n = 134), San Agustin, Junin (field2, n = 148) and Acos, Pasco (field3, n = 117). Field1 trial was conducted in the central coastal desert between July 12 and November 15, 2011, while field2 and field3 trials were conducted in the central highlands from September 7, 2011 to January 20, 2012 and June 12 to November 8, 2012, respectively. All sites are located in Peru. Detailed information on soil type and climate in the trial sites is given in Table 6.

**Table 6:** Detailed geographical location of the field trials and the characteristics of the soil and climate data recorded from the sites

Trial	Province	Locality	Altitude masl	Latitude South	Longitude West	Soil analysis				Temperature °C		Relative humidity %		Total precipitation mm
						S-S-C^a^ %	OM^b^ %	pH	EC^c^	Range	Mean	Range	Mean	
Field1	Lima	La Molina	244	−12.076289	−76.948417	57-25-15	1.3	7.4	4.3	12-25	16.0	70-91	85	<1
Field2	Junin	San Agustin	3265	−12.029167	−75.244167	39-31-30	1.6	7.5	0.4	1.6-30	12.5	12-100	78	332
Field3	Pasco	Acos	2612	−10.766444	−75.738361	66-16-18	1.9	4.9	0.7	2.9-24	13.5	14-91	72	290

^1^ sand-silt-clay

^2^ organic matter

^3^ electric conductivity mmhos/cm

In field1 and field2 trials, *in vitro* plants were first planted in Jiffy™ strips and after 15 days they were planted in the field using an alpha lattice design [48] with two replications. Each genotype was represented by ten plants in each of the replicates. In the trial field3, second generation tubers were planted using the same design, but with three replications and 15 plants per genotype per replicate.

In all field trials the planting distance was 0.9 m between rows and 0.3 m between plants. All trials were irrigated: field1 using irrigation canals, field2 and 3 using sprinklers. The fields were fertilized at planting by applying 160 kg/ha potassium chloride (KCl), 180 kg/ha phosphate ((NH4)_2_ HPO_4_), and 100 kg/ha nitrogen (CO(NH_2_)_2_). Nitrogen was applied a second time at the same dosage during the first hilling. Tubers were harvested when more than 75 % of the plants had reached foliar senescence.

### Phenotypic evaluation and statistical analysis

After harvest, tubers were washed, and fully developed tubers were selected for phenotypic evaluation. The general tuber shape was scored based on visual examination according to the morphological descriptors of Ortiz and Huaman [16], and each genotype was given a shape classification based on the average tuber shape. The scale consists of the following eight categories based on length and width at different parts of the tuber: compressed tubers are slightly wider than long; round and ovoid tubers are more long than wide and have a narrower apex (rose end) than base (heel end); obovoid tubers are longer than wide and have a narrower base; elliptic tubers have significantly narrower apex and base than the middle; oblong tubers have similar width at all parts of the tuber and are at maximum twice as long as wide; long-oblong tubers also have similar width at all parts of the tuber and their ratio of tuber length to width is between two and three; and elongated tubers are more than three times as long as wide. Eye depth was estimated by visual examination and classification into four classes: protuberant, superficial (<2 mm), slightly deep (2-4 mm), deep (5-6 mm) and very deep (>6 mm).

Tuber shape was also measured as the ratio of tuber length to width by dividing the length (mm) of the tuber from the base to the apex, by the width (mm) at the middle of the tuber. For each genotype, the average of the ratio of tuber length to width from three tubers was used as the trait value. For tubers that clearly had a narrower base and/or apex as compared to the middle, the width was also measured at the apex and the base. These measurements served for reinforcing the classification according to the above mentioned eight categories. Basic and multivariate analysis was performed in ‘R’ [49]. Both qualitative and quantitative data were checked for outliers and distribution.

### Genetic map construction and QTL analysis

Separate parental maps of ‘DMDD’ population were constructed using marker data previously developed by the Potato Genome Sequencing Consortium (PGSC) to construct genetic and physical maps for ‘DMDD’ [18]. Genetic maps were established for each parent separately as described by Khan et al.[23]. Briefly, map positions for all markers were extracted from the published map [18] and all markers with incomplete information (heterozygous in both parents and two alleles segregating) were excluded from the analysis. Markers that were heterozygous in the female parent and homozygous in the male parent were used to construct the maternal map, while the markers that were heterozygous in the male parent and homozygous in the female parent, were used to construct the paternal map. The map positions corresponding to [18] were used for parental maps after subtracting the map position of the first marker in cases where it was not ‘0’. Therefore, the map positions, order of markers and interval between the markers is as presented by Sharma et al. [18]. Mapping was done using the ‘CP’ option of JoinMap® 4 [50]. Phenotypic data for each experiment and male- female based genetic maps were used for QTL analysis using default options for interval mapping (IM) in MapQTL® 6 [51]. Kruskal-Wallis test was performed for the categorical variables using default options in MapQTL® 6 [51], while quantitative data were analyzed using interval mapping. A LOD threshold was determined using permutation test with 1000 permutations to declare a QTL genome-wide significant at 99 % significance threshold. The marker toPt-437059 with the highest LOD value was used to validate the trait-QTL association. The progeny individuals were grouped according to the marker genotype (nn or np) to compare the phenotypic data between the two groups.

### *In silico* identification of candidate genes

Gene lists were extracted from the pseudomolecule within a region of approximately +/− 1 cM of the significant QTLs, corresponding to +/− 1.31 Mb, +/− 1.25 Mb and+/− 2.17 Mb for chromosomes 10, 12, and 5, respectively. Gene lists were generated using the DM pseudomolecule AGPv4.3 build [18] and gene annotation v3.4 (http://solgenomics.net/organism/Solanum_tuberosum/genome) [28].

## Additional file

Additional file 1: Table S1. Gene lists were extracted from the pseudomolecule within a region of approximately +/− 1 cM of the significant QTLs. Gene lists were generated using the DM pseudomolecule AGPv4.3 build (Sharma et al. 2013) and gene annotation v3.4 (http://solgenomics.net/organism/Solanum_tuberosum/genome) (PGSC). Genes highlighted in bold have been specifically mentioned in the main manuscript. Genes highlighted in bold and italic are closer to the QTL markers.
